# Fighting the fakes

**DOI:** 10.1002/nop2.210

**Published:** 2018-10-09

**Authors:** Roger Watson

Earlier this year, in July, I co‐presented a seminar in Melbourne, Australia, with Professor Philip Darbyshire, Adelaide University and Professor Linda Shields, Charles Sturt University, Australia, with the title “Fighting the Fakes” which, as you will have guessed, was dedicated to our experiences with predatory publishers. The conference was the Annual Sigma International Nursing Research Conference, and we were given the early morning slot on the final half day of the conference. This is not usually a “crowd‐puller” but we had an audience of 40 people and, having left plenty of time for questions and answers, were inundated with comments, questions and several calls to action expressed as “what can we do?”.

In fact, our seminar was really about what we can do and foremost amongst our points was that we must raise awareness of the existence of predatory publishers and then, lest people forget, maintain that awareness. We need to keep raising awareness because it is clear many people are still unaware, and a proportion of those people fall for the enticing, obsequious and flattering emails with which we are bombarded daily. If only one academic a week is sending a manuscript to a predatory publisher in each university across the world then they are in receipt of millions of dollars annually. We need not doubt the evidence for, largely, it is there for all to see–a great deal of the manuscripts sent to predatory publishers do get published. Without something to show for their activities, then they are unlikely to continue to entice people. But it must be emphasized that manuscripts published by predatory publishers are worthless; they have not been peer‐reviewed, and no respectable university will accept them as credible publications for promotions or job applications. However, beyond this as explained previously in these editorials (Watson, 2017, 2018 ), there is other fraudulent activity such as fake or hijacked websites which will take your money and publish nothing and then the use–without their consent–of the names of respectable academics to festoon editorial boards and even, allegedly, to edit journals. People must be under no illusions–this is a fraudulent and criminal activity, and we have no way of knowing to what use the profits are put but just imagine money laundering, drug‐dealing and illegal arms purchases. One naive soul once replied to me on Twitter reckoning that these were simply poor people in developing countries trying to make a living. First, what difference would that make–even if it were true–and then could the same not be said of the same poor people selling their daughters into the sex industry or processing narcotics for sale on our streets?

So, as to what can be done–it seems that raising awareness is the mainstay of any campaign. Stopping this attempt at fraudulent trade in our ideas, intellectual property and reputations seems impossible unless we starve it of funds. When the effort over reward ratio increases and profits falter, they'll stop. Meantime, any strategies to stop the deluge of predatory emails–including invitations to predatory conferences–seem impossible. You can delete, ignore, spam, correspond (don't, they only think you're interested), unsubscribe (which is simply an illusion) and protest; nothing works. The predators have an endless combination of bizarre and hybrid names that they can combine in virtually endless combinations before they find a way to your inbox again. Currently, all we can do is delete on sight.

Beyond that, we need to be vigilant personally, share our experiences and hope that our universities will be more explicit with staff about the consequences of predatory publishing and introduce some sanctions. Beyond that, universities and other responsible bodies should begin to take a lead and indicate where it is safe to publish and provide approved lists of journals. This is not an impossible task, and once initiated, journals will be scrambling to have themselves included on these lists and, to avoid exclusion, will be ensuring that the things that prove their bona fides: PubMed and Scopus listing; Clarivate Analytics; Directory of Open Access Journal (DOAJ) and many others are prominent, genuine and demonstrable (*Nursing Open* is listed on the DOAJ and has recently achieved Scopus listing).
